# Probing the Characterization of the Interaction of Aflatoxins B1 and G1 with Calf Thymus DNA In Vitro

**DOI:** 10.3390/toxins9070209

**Published:** 2017-07-01

**Authors:** Liang Ma, Jiaman Wang, Yuhao Zhang

**Affiliations:** College of Food Science, Southwest University, Beibei District, Chongqing 400715, China; wangjiaman2412@163.com (J.W.); zhy1203@163.com (Y.Z.)

**Keywords:** aflatoxins, interaction, binding mode, molecular modelling

## Abstract

The binding characterization of aflatoxins with calf thymus DNA (ctDNA) under physiological conditions was investigated. Multispectroscopic techniques, ctDNA melting, viscosity measurements, and molecular docking techniques were employed to elucidate the binding mechanism of the aflatoxins with DNA. The fluorescence results indicated that both aflatoxin B1 (AFB1) and aflatoxin G1 (AFG1) bound to the ctDNA, forming complexes through hydrogen bonding. The binding constants of AFB1 and AFG1 with ctDNA reached up to 10^3^ L·mol^−1^ and 10^4^ L·mol^−1^, respectively, and AFG1 exhibited a higher binding propensity than that of AFB1. Furthermore, both AFB1 and AFG1 bound to the ctDNA through groove binding, as evidenced by the results of the spectroscopic, iodide quenching effect, viscosity, and ctDNA melting measurements. Changes in the circular dichroism signal manifested that both AFB1 and AFG1 induced an increase in the right-handed helicity, but only minimally influenced the base stacking of the DNA. A molecular docking study of the aflatoxin’s binding with the DNA revealed a groove binding mode, which was driven mainly by hydrogen bonding. This study of aflatoxin–ctDNA interaction may provide novel insights into the toxicological effect of the mycotoxins.

## 1. Introduction

**Figure 1:**
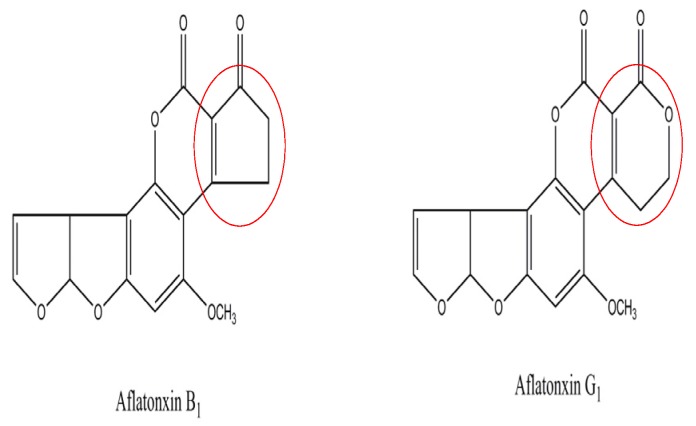
Molecular structures of Aflatoxin B1(AFB1) and Aflatixin G1(AFG1).

Aflatoxins are among the most toxic mycotoxins and have attracted considerable attention because of the great threat they pose to human health. The aflatoxin contamination of animal feeds and food has become an important issue because of the potential adverse health effects of aflatoxin, including kidney disorders, immune suppression, and even cancers [1]. The exposure to aflatoxins has adverse effects on the animal and food industries [2]. Aflatoxin B1 (AFB1) and aflatoxin G1 (AFG1) (Figure 1) are secondary toxic metabolites mainly produced by the fungi *Aspergillus flavus* and *Aspergillus parasiticus*, and commonly contaminate peanut, corn, cotton and other oil crops [3].

AFB1 is one of the most toxic mycotoxins and has been classified as a group I known human carcinogen [4]. It also displays other biological effects such as mutagenicity and teratogenicity in humans and animals [5]. Moreover, the International Agency for Research on Cancer has classified AFG1 as a group 2B toxin, indicating that it is possibly carcinogenic to humans [6]. AFG1 possesses a similar structure to that of AFB1, and AFG1 toxicity is known as second only to AFB1 toxicity among all the aflatoxins. AFG1 has also induced liver-tumor formation in experimental animals, but usually at a lower occurrence than that of AFB1 [7].

DNA is an important macromolecule that carries substantial hereditary information, controls cellular metabolism, and facilitates the biological synthesis of protein [8]. DNA has been proven as a target molecule of various drugs, pesticides, and toxic compounds [9,10]. Pesticides and other harmful substances could enter cells and interact with the DNA, which may cause a change in the DNA, and even lead to genetic mutations or the occurrence of cancer [11,12]. Therefore, exploring the interaction mechanisms of aflatoxins with DNA is of great importance for selecting therapeutic drugs and understanding DNA damage mechanisms. Generally, small molecules interact with DNA through covalent or noncovalent interactions. Additionally, small molecules interact with DNA in a noncovalent binding through three modes: (i) intercalation between the base pairs; (ii) groove binding; and (iii) electrostatic interaction [13]. Among the three binding modes, intercalative binding is the most effective, and causes the greatest damage on the DNA’s conformation. AFB1 is metabolized as AFB1–exo-8,9-epoxide by human cytochrome P450s after entering the body. AFB1–exo-8,9-epoxide can bind covalently to DNA to form the AFB1–DNA adduct [14]. The formation of the AFB1–N7−Gua adduct has been proven to be related to the occurrence of hepatic tumors in trout and rats [15].

However, a portion of aflatoxins can directly interact with DNA in cells without being metabolized as their epoxides. In this case, a reversible noncovalent interaction ensues between the aflatoxins and the DNA. To some extent, owing to the reversibility of noncovalent interactions, it is typically preferred over covalent adduct formation when keeping the drug metabolism and toxic side effects in mind [16]. In our study, the noncovalent interactions of two kinds of aflatoxins with calf thymus DNA (ctDNA) were investigated in vitro. UV–vis spectroscopy, fluorescence spectroscopy, circular dichroism (CD), and molecular docking were employed to study the interaction mechanisms of aflatoxins with DNA. Competitive displacement, iodide quenching effect, viscosity, and DNA melting assays were used to identify the binding mode. We hope that this study will be helpful to further an understanding of the binding mechanisms of aflatoxins with DNA and the toxicological effects of aflatoxins.

## 2. Results

### 2.1. Dialysis Assay

**Figure 2:**
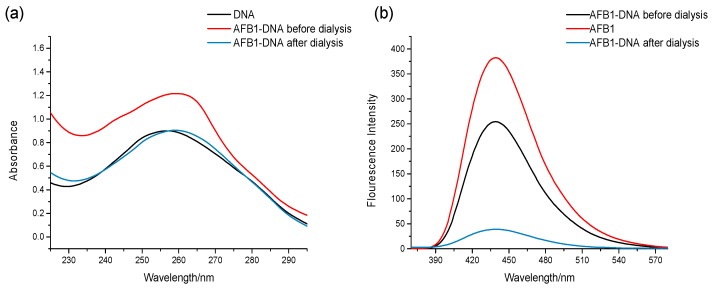
UV Absorption (**a**) and fluorescence emission (**b**) spectra of the AFB1-DNA before and after the dialysis. c_(DNA)_ = 1.51 × 10^−4^ mol·L^−1^, c_(AFB1)_ = 1 × 10^−5^ mol·L^−1^ (*n* = 3).

Aflatoxins can bind to DNA via covalent interactions (irreversible binding) or noncovalent interactions (reversible binding). Hence, we initially explored the interaction mode of the binding between AFB1/AFG1 and DNA. In this study, dialysis was employed to investigate the binding mode between AFB1/AFG1 and DNA [16]. If the bind between AFB1/AFG1 and the DNA was noncovalent, the UV absorbance and fluorescence emission of the AFB1/AFG1–DNA complex would be changed after the dialysis because of the separation of the small molecules from the DNA. This could be attributed to the reversibility of the interaction. However, owing to the fact that covalent binding is irreversible, if the combination of AFB1/AFG1 and DNA were covalent, the UV absorbance and fluorescence emission of the AFB1/AFG1–DNA complex would not be significantly altered after the dialysis. Therefore, we studied the changes in the UV absorbance/fluorescence spectra of the AFB1–DNA and AFG1–DNA complexes in this manner. Figure 2 shows whether the absorbance or emission of the AFB1–DNA complex had changed after dialysis. The reason for the change in spectra was that AFB1 was separated from the DNA. The absorbance and emission spectra changes of the AFG1–DNA complex were in agreement with that of the AFB1–DNA complex. Hence, the interaction between AFB1/AFG1 and ctDNA was noncovalent rather than covalent.

### 2.2. Fluorescence and Resonance Light Scattering (RLS) Spectra

#### 2.2.1. Fluorescence Studies

Given that aflatoxins could emit fluorescence under ultraviolet irradiation, a series of experiments based on fluorescence spectroscopy were performed to explore the binding mechanism of aflatoxins to DNA. AFB1/AFG1 displayed strong emission spectra, with emission maxima at 440 nm when excited at 365 nm. The fluorescence intensity of AFB1/AFG1 consistently decreased with increasing amounts of ctDNA. Moreover, the quenching rates of AFB1/AFG1 were 37% and 61.5% when the DNA concentration reached 5 × 10^−5^ mol·L^−1^, whereas the position and shape of the peaks did not change. Thus, AFB1/AFG1 interacts with ctDNA through noncovalent bonds rather than covalent bonds [17]. This finding indicates that DNA is a potential target of small molecules and directly confirms the interaction between AFB1/AFG1 and ctDNA [18].

The common types of fluorescence quenching include static quenching, dynamic quenching, and the combination of both. Therefore, fluorescence data could be studied using the Stern–Volmer equation [19]:

(1) $$\frac{F_{0}}{F}=1+K_{q}\tau_{0}\left[Q\right]=1+K_{sv}\left[Q\right]$$

where *F*_0_ and *F* are the fluorescence intensities in the absence and presence of a quencher; *K_q_* is the quenching rate constant of the biomolecules; *τ_0_* is the average lifetime of the biomolecule without a quencher (1 × 10^-8^ s); *K_sv_* is the Stern–Volmer quenching constant; and [*Q*] is the quencher’s concentration [20].

**Figure 3:**
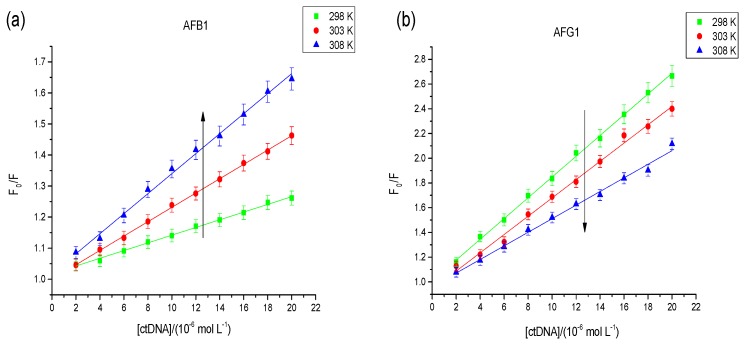
Stern–Volmer plot for observed fluorescence quenching of AFB1 (**a**) and AFG1 (**b**) upon addition of DNA at different temperatures. c_(AFB1)_ = 8 × 10^−6^ mol·L^−1^. c_(DNA)_ = 1.6, 3.2, 4.8, 6.4, 8, 9.6, 11.2, 12.8, 14.4, 16 × 10^−6^ mol·L^−1^ (*n* = 3). “ctDNA” means calf thymus DNA.

Dynamic quenching depends on diffusion, that is, a temperature rise will increase the effectiveness of the collision molecule, thus *K_sv_* will be increased. Static quenching occurs when ground-state complexes are formed by the binding of fluorophore and quencher molecules [21]. In contrast, the stability of ground-state complexes will be reduced with an increased temperature, thus *K_sv_* will be decreased. As shown in the Figure 3, the type of quenching interaction between AFG1 and the DNA was static quenching; however, both static and dynamic quenching was found between AFB1 and the DNA.

The binding constant *(K_a_*) for the complex formed between AFB1/AFG1 and ctDNA was estimated using modified Stern−Volmer equation [22]:

(2) $$\frac{F_{0}}{F_{0}-F}=\frac{1}{f_{a}K_{a}}\frac{1}{\left[Q\right]}+\frac{1}{f_{a}}$$

where *f_a_* is the fraction of accessible fluorescence, and *K_a_* is the modified Stern−Volmer association constant for the accessible fluorophores. The constant *K_a_* is the quotient of an ordinate 1/*f_a_* and slope 1/*f_a_K_a_*. The corresponding *K_a_* values at three different temperatures are displayed in Table 1. The altered trend of *K_a_* with rising temperature agrees with the trend of *K_sv_* analyzed previously. This result indicates that the binding of AFB1/AFG1 with DNA was feasible, and that a reversible AFB1/AFG1–DNA complex may have formed [23]. The *K_a_* value of the AFB1–DNA complex at 298 K was 4.12 × 10^3^ L·mol^−1^, which agrees with that obtained through subsequent UV spectroscopy and supports the effective role of static and dynamic quenching [24]. A comparison shows that the *K_a_* of the AFG1–DNA complex was greater than that of the AFB1–DNA complex. The difference in *K_a_* mainly depends on the structural differences of the aflatoxins. This indicates that the hexatomic ring of AFG1 has a larger dimension and stronger stability than the five-member ring of AFB1 and that induced AFG1 has a stronger DNA binding than AFB1.

The corresponding thermodynamic parameters were analyzed to further probe the characteristics of the interaction between aflatoxins and DNA. Several binding forces exist between small molecules and biological macromolecules, including electrostatic interactions, van der Waals interactions, hydrogen bonds, and hydrophobic forces. The thermodynamic parameters were calculated using the following equations to explore the acting forces [25]:

(3) $$LnK=-\frac{\Delta H}{RT}+\frac{\Delta S}{R}$$

(4) ΔG=ΔH−ΔT⋅S

where *R* refers to the gas constant (8.314 J·K^−1^·mol^−1^), T refers to the temperature in Kelvin, and *K* is the binding constant at different temperatures (298, 303, and 308 K). Δ*H* can be considered a constant when the temperature varies in small degrees. According to the perspective of Ross and Subramanian, the main interaction force can be concluded as follows: (1) hydrophobic interactions, Δ*H* > 0 and Δ*S* > 0; (2) van der Waals or hydrogen bonds, Δ*H* < 0 and Δ*S* < 0; and (3) hydrogen bonding with hydrophobic interactions, Δ*H* < 0 or Δ*H* ≈ 0 and Δ*S* > 0 [25]. As shown in Table 2, the hydrogen bonding with hydrophobic interaction played a major roles in the binding between AFB1 and the DNA and contributed to the stability of the complex. Unlike AFB1, the negative values of Δ*H* and Δ*S* indicate that the AFG1–DNA complexation process was driven mainly by hydrogen bonds and van der Waals forces. Moreover, the two kinds of binding reaction were also spontaneous as indicated by the negative signs of the Δ*G* values [25].

#### 2.2.2. RLS Spectral Analysis

**Figure 4:**
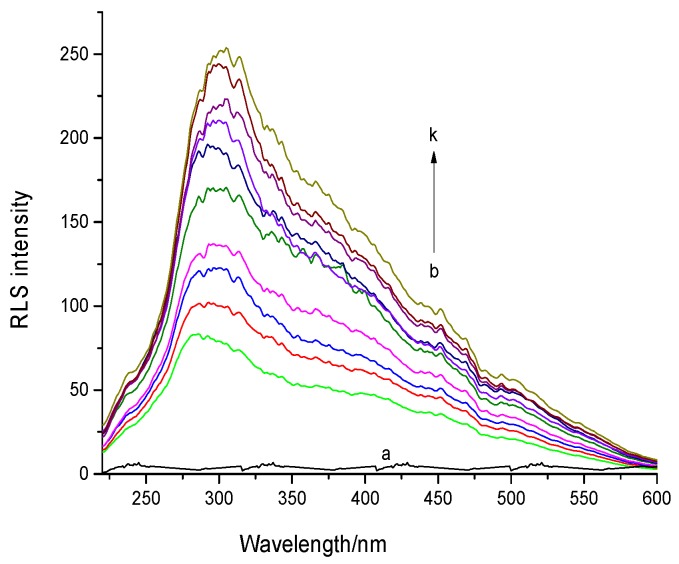
Resonance light scattering (RLS) spectra of DNA-AFB1 system at pH 7.4 and 298 K. Curves: (a) c_(AFB1)_ = 0 and c_(DNA)_ = 14.4 × 10^−6^ mol·L ^−1^; (b–k) c_(DNA)_ = 0, 1.6, 3.2, 4.8, 6.4, 8, 9.6, 11.2, 12.8 and 14.4 × 10^−6^ mol·L^−1^, respectively, and c_(AFB1)_ = 8 × 10^−6^ mol·L^−1^ (*n* = 3).

The RLS spectra of AFB1 and the AFB1−ctDNA system in the phosphate-buffered saline (PBS) buffer (pH 7.4) are shown in Figure 4. The RLS intensity of AFB1 was significantly increased with the increasing of the ctDNA concentration, which indicated that AFB1 interacted with the DNA. According to RLS theory, RLS intensity is related to the dimension of the formed particle, that is, larger particles would produce stronger light-scattering signals [26,27]. Therefore, we concluded that the formation of an AFB1-DNA complex was due to the addition of DNA. Based on the results of previous related studies, we assumed that the enhancement of the RLS signal of AFB1was due to the groove binding of the ctDNA rather than intercalation [9,28]. Further experiments were performed to confirm this conclusion.

### 2.3. Competitive Displacement Assays

**Figure 5:**
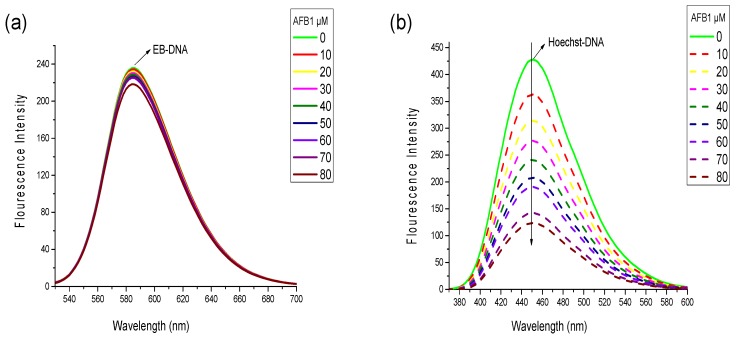
Fluorescence emission spectra of the competitive displacement assays between AFB1 and different dyes. (**a**) Emission spectra of the EB-DNA complexes in the present of AFB1. c_(EB)_ = 2 × 10^−6^ mol·L^−1^, c_(DNA)_ = 4 × 10^−5^ mol·L^−1^, c_(AFB1)_ = 0, 1, 2, 3, 4, 5, 6, 7, 8 × 10^−5^ mol·L^−1^; (**b**) Emission spectra of the Hoechst-DNA complexes in the present of AFB1. c_(Hoechst)_ = 2 × 10^−6^ mol·L^−1^, c_(DNA)_ = 4 × 10^−5^ mol·L^−1^, c_(AFB1)_ = 0, 1, 2, 3, 4, 5, 6, 7, 8 × 10^−5^ mol·L^−1^ (*n* = 3).

To identify the binding mode of AFB1/AFG1 to the DNA from various aspects, competitive displacement experiments were performed. Considering that any small molecule replacing the binding dye, such as EB and Hoechst 33258, from DNA will interact with the DNA via the same mode as the replaced dye, the binding mode of AFB1/AFG1 could be elucidated by studying the changes in fluorescence spectra of a dye–DNA complex with the addition of aflatoxins [29]. EB used as a fluorescence probe and could bind to DNA via intercalation [30]. Notably, neither a shift nor quenching of the fluorescence peak of EB was observed when AFB1/AFG1 was added, suggesting that no interaction existed between AFB1/AFG1 and EB. Similar results were observed with Hoechst 33258. Free EB has very weak fluorescence intensity in a PBS buffer. However, the fluorescence intensity of EB increased when the planar ring of EB was inserted between base pairs in the DNA double helix [30]. The fluorescence spectra of the EB–ctDNA complex in the absence and presence of AFB1 are shown in Figure 5a. With increasing AFB1 concentration, the emission intensity of the EB–DNA complex’s slightly decreased and did not significantly change. The final fluorescence intensity reached 90% of the original EB–DNA fluorescence intensity with the addition of AFB1, indicating that AFB1 did not replace the EB from the DNA’s double helix.

Hoechst 33258 is a classic groove-binding dye that binds in the minor groove of DNA [31]. Similar to EB, Hoechst 33258 can produce intense fluorescence through when it binds to DNA through groove binding. At this point, we observed an apparent fluorescence quenching effect different from that in Figure 5b. With the addition of AFB1, the fluorescence intensity of the Hoechst 33258–DNA solution remarkably decreased at 457 nm because many Hoechst 33258 molecules were replaced from the DNA‘s groove and released into the solution. AFG1 caused a similar reduction in the fluorescence intensity of the EB/Hoechst 33258-ctDNA complex. Given the above results, we inferred that groove binding is the major binding mode between AFB1/AFG1 and ctDNA.

### 2.4. UV–Visible Spectroscopy

**Figure 6:**
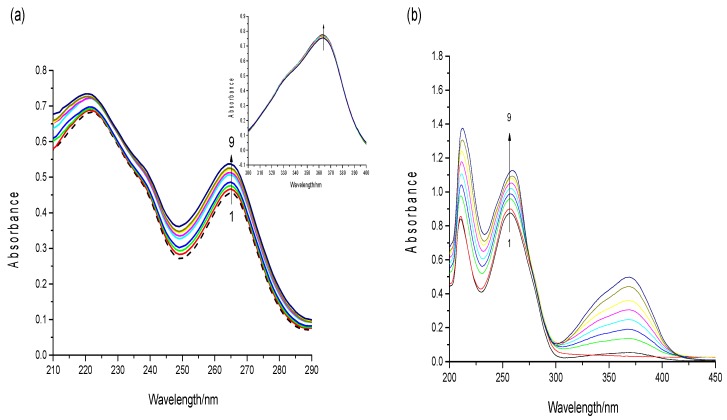
(**a**) UV-visible absorption spectra of AFB1 (3.5 × 10^−5^ mol·L^−1^) in the presence of increasing concentrations of ctDNA in in a phosphate-buffered saline (PBS buffer (pH 7.4). c_(DNA)_ = 0, 3.2, 6.4, 9.6, 12.8, 16.0, 19.2, 22.4, 25.6 × 10^−6^ mol·L^−1^ (from 1 to 9). Insert: fluorescence intensity at 365 nm; (**b**) UV-visible absorption spectra of the DNA (2 × 10^−5^ mol·L^−1^) in the presence of increasing concentrations of AFB1 in PBS buffer (pH 7.4). c_(AFB1)_ = 0, 4, 8, 12, 16, 20, 24, 28, 32 × 10^−6^ mol·L^−1^ (from 1 to 9) (*n* = 3).

DNA has a characteristic absorption peak at approximately 260 nm, which is caused by aromatic base and phosphate chromophores [32]. To thoroughly observe the spectrum changes, a high concentration of aflatoxins (3.5 × 10^−5^ mol·L^−1^) was used in a UV–vis absorption experiment. The UV spectra of the AFB1 solution as ctDNA was added was obtained. As shown in Figure 6a, with an increasing concentrations of ct-DNA, AFB1 exhibited significant hyperchromism with slight blue shift at 267 nm and 221 nm. However, the absorption peak of AFB1 at 365 nm revealed only slight hyperchromism. The peak at 365 nm was a characteristic peak of the AFB1 molecule, which related to the toxicity of AFB1. Additionally, the peaks at 267/221 nm were attributed to the bifuran structure of AFB1. Based on this result, we inferred that DNA binding greatly influenced the bifuran structure of AFB1. Similar results were noted on the behavior of ctDNA in the presence of AFG1.

Moreover, the UV–vis spectra of the DNA with the addition of AFB1 are shown in Figure 6b. The absorption peak of DNA at 260/210 nm exhibited a gradual increase and red shift with the increasing concentration of AFB1. The hyperchromicity may be attributed to external contact (groove or electrostatic binding) or the disruption of the DNA’s secondary structure [33]. Also, the absence of isobestic points in the UV–vis spectroscopy indicated that the complex formations had noncovalent interactions, such as groove binding [34]. These results indicate that the binding of AFB1 to ctDNA is nonintercalating, which is possibly caused by hydrophobic forces or hydrogen bonding of the oxygen atoms in AFB1 with the DNA’s nucleobases [35]. The phenomena observed with increasing AFG1 concentration were similar to those observed for AFB1.

For a qualitative determination of the interaction intensity between AFB1/AFG1 and ctDNA, the binding constants (*K_b_*) were calculated using the following equation [36]:

(5) $$\frac{A_{0}}{A-A_{0}}=\frac{\varepsilon_{G}}{\varepsilon_{H-G}-\varepsilon_{G}}+\frac{\varepsilon_{G}}{\varepsilon_{H-G}-\varepsilon_{G}}\cdot \frac{1}{K_{b}\left[DNA\right]}$$

where *A*_0_ and *A* are the absorbance of AFB1/AFG1 in the absence and presence of DNA, and *ε_G_* and *ε_H–G_* are their absorption coefficients respectively. *G* and *H*–*G* represents the ctDNA and AFB1/AFG1–DNA species. In the plot of *A*_0_/(*A* − *A*_0_) versus 1/[*DNA*], *K_b_* was obtained by obtaining the intercept-to-slope ratio. The *K_b_* value of the AFB1–DNA complex was found to be 4.26 × 10^3^ L·mol^−1^, which was less than that of the AFG1–DNA complex (*K_b_* = 1.43 × 10^4^ L·mol^−1^). The calculated *K_b_* values at 298 K (order of 10^3^ or 10^4^) were smaller than that reported for a typical classic intercalator (order of 10^5^) [37]. Furthermore, by comparison with the *K_b_* value of other DNA groove binders, the binding mode between AFB1/AFG1 and the DNA was deduced to be groove binding [38].

### 2.5. Viscosity Measurements

**Figure 7:**
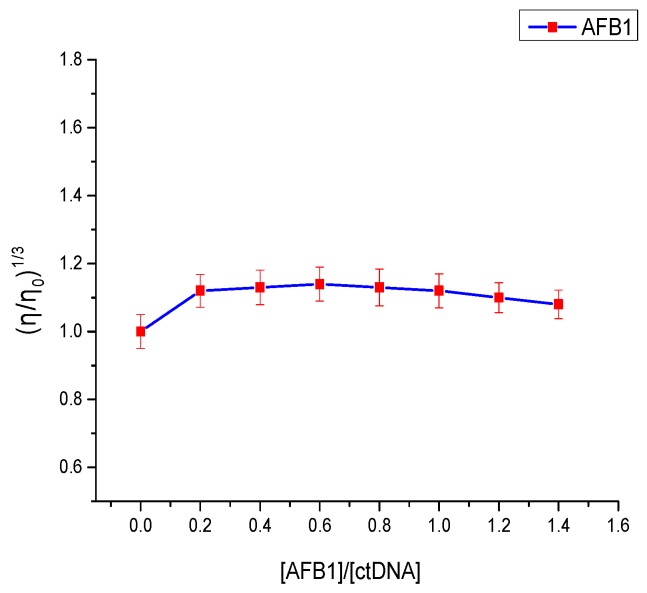
Effect of the addition of AFB1 on the relative viscosity of ctDNA at 298 K. c_(DNA)_ = 2 × 10^−5^ mol·L^−1^, [AFB1]/[ctDNA] = 0, 0.2, 0.4, 0.6, 0.8, 1, 1.2, 1.4 (*n* = 3).

Viscosity experiments are regarded as the least ambiguous and effective tool to explore the binding mode of small molecules to DNA [39]. Intercalation binding increases DNA viscosity because small-molecule intercalation requires a sufficiently large space between base pairs to accommodate the bound molecules and elongate the double-helix DNA [40]. In contrast, few changes were observed on the DNA’s viscosity when electrostatic interaction or groove binding ensued during the binding process [41]. As seen in Figure 7, the viscosity of the DNA solution showed no significant change upon the addition of AFB1. Similarly, such change was exhibited in the binding of AFG1 to the DNA. This proved that the binding mode of AFB1/AFG1 to ctDNA is groove binding.

### 2.6. DNA Melting Assay

**Figure 8:**
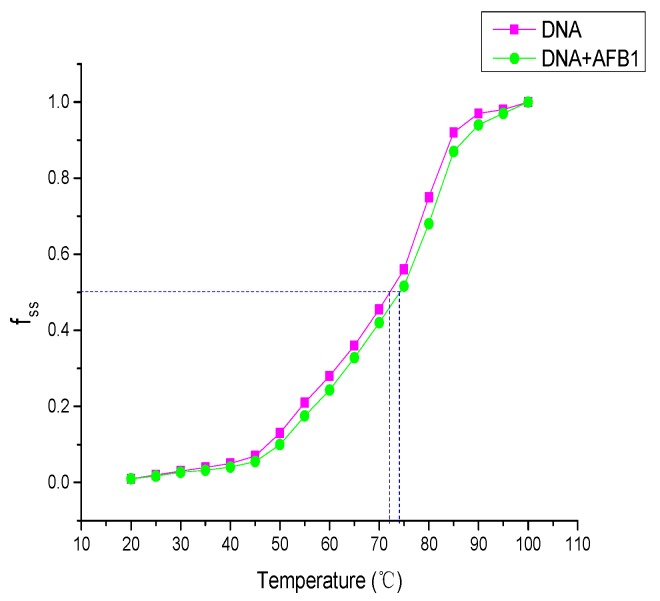
Melting curves of ctDNA at 260 nm in the absence (pink) and the presence (green) of the various concentrations of AFB1 in the PBS buffer. c_(DNA)_ = 8 × 10^−6^ mol·L^−1^, c_(AFB1)_ = 8 × 10^−6^ mol·L^−^^1^ (*n* = 3).

The thermal melting temperature (*T*_m_) is the temperature at which half of a DNA sample melts and it reflects the stability change of the DNA double-helix structure [29]. Thermal melting temperature is also an important parameter for determining the binding mode of small molecules to DNA [42]. Small-molecule interaction with DNA could influence *T*_m_. Generally, intercalative binding could stabilize the DNA helix structure and increase the Tm of the DNA by approximately 5–8 °C, whereas nonintercalation binding, such as groove or electrostatic binding, along the phosphate backbone of DNA causes small changes in the *T*_m_ [43]. The melting curves of the DNA in the absence and presence of AFB1 are shown in Figure 8. As shown, the Tm of the DNA in the absence of AFB1 was 73 ± 1 °C. Meanwhile, the observed *T*_m_ of DNA in the presence of AFB1 was 74 ± 1 °C. These results clearly indicate that AFB1’s addition has minimal to no effect on the Tm of DNA. Hence, we concluded that the interaction of AFB1 with DNA did not increase the stability of the DNA’s double-helix structure. A similar change was observed in the binding of AFG1 to DNA. When AFG1 was added to DNA solution, the *T*_m_ of the DNA increased from 73 ± 1 °C to 75 ± 1 °C. The results provided further evidence for the nonintercalative binding between AFB1/AFG1 and DNA.

### 2.7. Effect of Ionic Intensity

To further distinguish the binding mode between aflatoxins and DNA, the effect of ionic intensity was employed as an efficient method. DNAs’ double helices are anionic polyelectrolytes with phosphate groups [34]. In this study, NaCl was used to control the ionic intensity of the systems. Given that Na+ was released to neutralize negative charges on the phosphate backbone of the the DNA when the NaCl dissolves, the electrostatic repulsion between the phosphate backbone was reduced, and the shrinkage degree of the DNA chains was gradually increased [44]. The ions would also compete for the binding sites between the small molecules and the phosphate backbone of the DNA; hence, the free concentration of the small molecules was increased in the solutions [44]. When the binding mode was electrostatic interaction, with the addition of NaCl, the fluorescence quenching degree of the small molecules by the DNA would attenuate the competition between the NaCl and the small molecules. 

**Figure 9:**
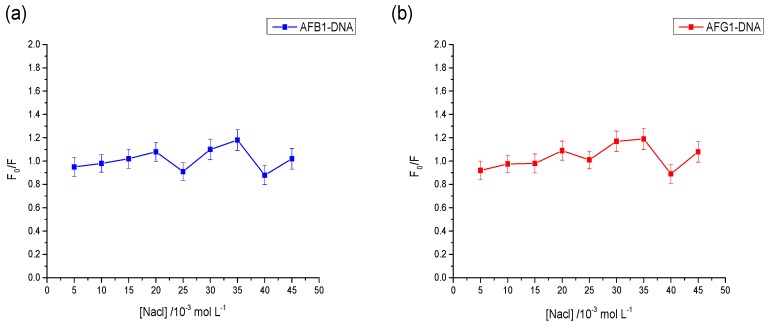
Effect of ionic strength on the fluorescence intensity of the AFB1-DNA complex (**a**) and the AFG1-DNA complex (**b**). c_(DNA)_ = c_(AFB1)_ = 2 × 10^−5^ mol·L^−1^, c_(NaCl)_ = 5, 10, 15, 20, 25, 30, 35, 40, 45 × 10^−3^ mol·L^−1^. *F*_0_ was the fluorescence intensity of AFB1/AFG1-DNA complex without the NaCl and *F* was the fluorescence intensity of AFB1/AFG1-DNA complex with the various concentrations of NaCl (*n* = 3).

To determine whether or not AFB1/AFG1 has an electrostatic binding with DNA, the effect of NaCl on the fluorescence of the AFB1–DNA and AFG1–DNA systems was studied. In Figure 9, *F*_0_ refers to the fluorescence intensity of aflatoxin–DNA systems in the absence of NaCl, and F corresponds to the fluorescence intensity of the aflatoxin–DNA systems in the presence of NaCl. As shown in Figure 9, after adding the DNA, no major changes in the fluorescence intensity were observed in either the AFB1–DNA complex or the AFG1–DNA complex. The results suggested that the interaction between AFB1/AFG1 and DNA excluded electrostatic interaction and groove binding would be predicted.

### 2.8. Effects of Aflatoxins on Native or Denatured DNA

**Figure 10:**
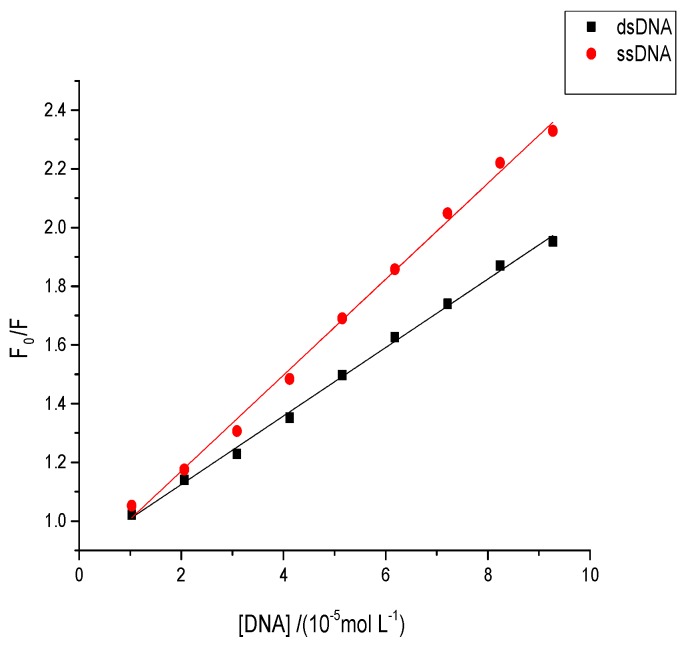
Fluorescence quenching plots of AFB1 by double-stranded DNA (dsDNA) and single-stranded DNA (ssDNA) at pH 7.4 and 298 K, c_(AFB1)_ = 5 × 10^−5^ mol·L^−1^. *F*_0_ was the fluorescence intensity of AFB1 without DNA and *F* was the fluorescence intensity of AFB1 with the various concentrations of ssDNA/dsDNA (*n* = 3).

A difference was observed in the quenching effect of single-stranded DNA (ssDNA) and double-stranded DNA (dsDNA) when small molecules bound with the DNA under different interaction modes. Under the grooving mode, the quenching effect of ssDNA should be stronger than that of dsDNA owing to the enlarged space groove, which allows binding between small molecules and DNA in the groove [45]. Conversely, a weaker quenching effect of ssDNA than dsDNA was noted when small molecules intercalated between the DNA base pairs because the double strands were denatured [45]. The quenching effects of both ssDNA and dsDNA also slightly differed when small molecules interacted with the DNA phosphate groups through an electrostatic binding mode [45]. The fluorescence quenching plots of AFB1/AFG1 upon the addition of different concentrations of dsDNA and ssDNA are shown in Figure 10. As shown, the ssDNA exhibited a greater slope than dsDNA in both AFB1 and AFG1. This also indicated that the *K_sv_* value of AFB1/AFG1 with ssDNA was larger than that with dsDNA. Moreover, the results suggest that the binding mode of AFB1/AFG1 to ctDNA might be groove binding. These results were in agreement with the above mentioned experiments.

### 2.9. Circular Dichroism Spectral Measurement

CD is a powerful and sensitive technique for monitoring the conformational alteration of DNA with the binding of exogenous substances [46]. CD signals are sensitive to the mode of DNA binding with the target, particularly within the 180–320 nm wavelength range [47]. Hence, a CD spectral study was conducted to detect the conformational change of DNA caused by the interacting aflatoxins. The CD spectra of the free DNA were characterized as two peaks, including a positive peak at around 275 nm (corresponding to the base-pair stacking) and a negative peak at around 245 nm ascribed to the right-handed helicity of B-DNA [48]. These two peaks are marker peaks of B-DNA assigned to the corresponding changes of DNA structure in the presence of small molecules. The intercalation mode changes the secondary structure of DNA, leading to significant alternation in the CD spectra of DNA, such as the changes in intensity and position of the peaks. Meanwhile, the groove binding and electrostatic interaction do not change CD spectra of the DNA because groove binding and electrostatic interaction have less or lack perturbation on the base stacking and helicity bands [49].

**Figure 11:**
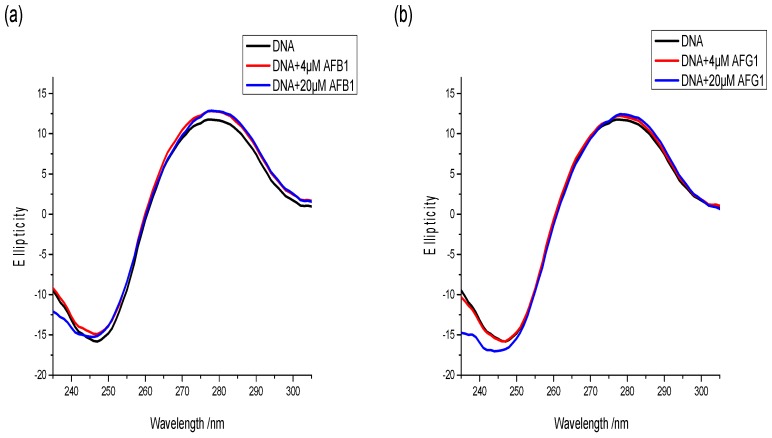
The change of circular dichroism (CD) spectrum of ctDNA in the absence and presence of AFB1 (**a**) and AFG1 (**b**), c_(DNA)_ = 1 × 10^−5^ mol·L^−1^, c_(AFB1)_ = c_(AFG1)_ = 4 × 10^−6^ mol·L^−1^ and 20 × 10^−6^ mol·L^−1^ (*n* = 3).

The changes in the CD spectra of the free DNA with increasing AFB1 and AFG1 concentrations were investigated and are shown in Figure 11. As shown, the intensities of the negative peak increased slightly. Moreover, the positive peaks were negligibly altered after AFB1/AFG1 binding. In addition, no significant shift was observed in the peak position regardless of AFB1 or AFG1 binding. Moreover, the binding of both aflatoxins to DNA could induce an increase in the right-handed helicity of DNA, but the B-DNA conformation was not destroyed [50]. This might be because AFB1/AFG1 bound in the minor groove region of DNA, making the double-helix structure of DNA to become loose [51]. Although a different effect on the intensity of CD spectra of DNA was noted with the binding of two aflatoxins, the same positive cotton effect on the AFB1–DNA and AFG1–DNA system was noted. This indicates that the effect of two aflatoxins on DNA conformation was similar, but only to varying degrees. With addition of AFG1, the intensity change of the negative peak was larger than that of AFB1, which indicated that AFG1 has a greater influence on the structure of DNA compared with AFB1. These conclusions are in accordance with the results from the fluorescence studies.

### 2.10. Molecular Docking Studies

**Figure 12:**
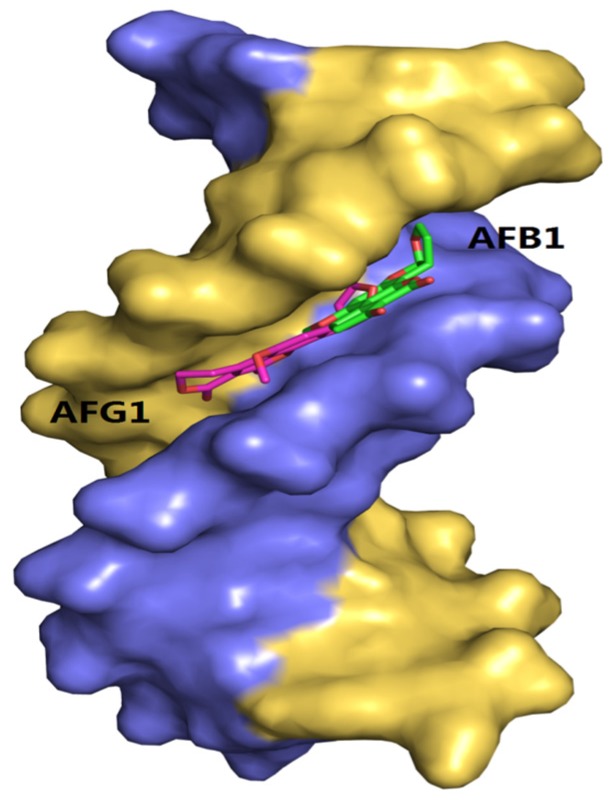
The molecular docked structures of AFB1/AFG1 binding to DNA through groove interaction. Different small molecules were represented by different colors: AFB1, green; AFG1, pink.

Molecular docking methods were originally used for drug design and are significantly important in structural molecular biology [52]. With the aid of docking software, we can predict the binding mode or binding sites of the interactions between ligands and macromolecules, and also obtain some information about the formation of energetically favorable complexes [53]. In addition, owing to the limitation of the theoretical methods, it should be associated with the other available experimental data [54]. In this study, the molecular docking analysis of the AFB1/AFG1–DNA complexes is shown in Figure 12. The results show that the binding modes of the AFB1/AFG1–DNA complexes were similar, that is, groove binding. The difference was that AFG1 bound with the A–T residues in the minor groove, paralleled to the trend of the double helix. Many groove binders with small dimensions were inclined to bind in the region of rich A–T base pairs, because this region was narrower than that of the rich C–G base pairs [55]

**Figure 13:**
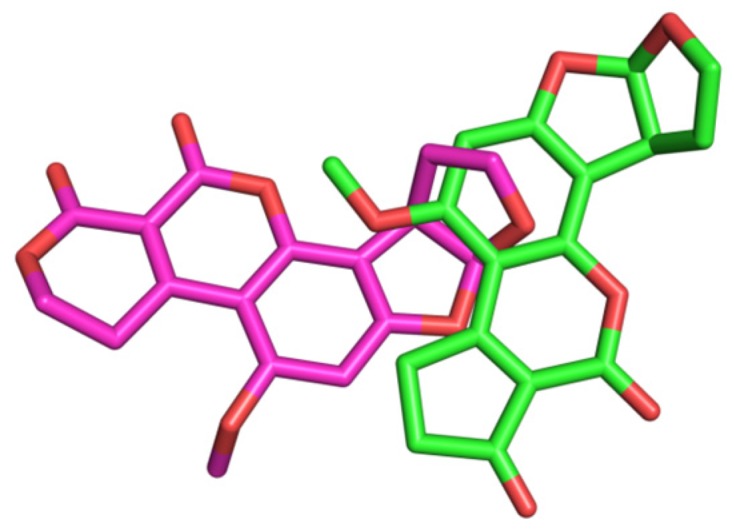
A comparison of binding conformation overlap between AFB1/AFG1 and DNA (AFB1: green; AFG1: pink).

**Figure 14:**
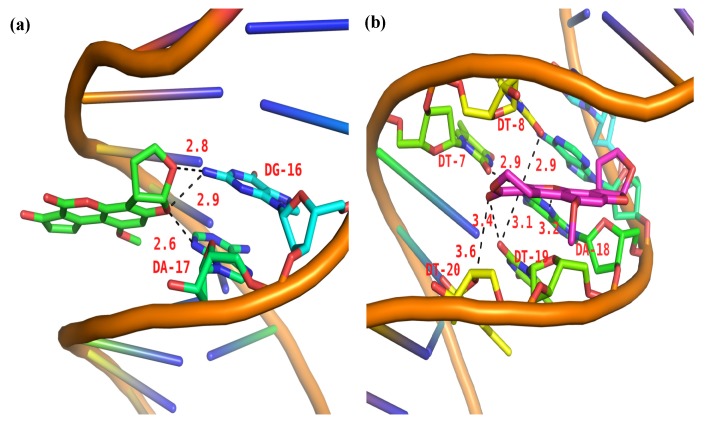
Most probable docking pose of AFB1 (**a**) and AFG1 (**b**) with ctDNA (possible hydrogen bonds shown as black dash).

The 100 docking results manifested that AFB1/AFG1 can bind with DNA with different energies. As shown in Table 3, AFG1 possessed the lowest binding energy of –8.29 kcal·mol^−1^, whereas that of AFB1 was –7.78 kcal·mol^−1^ when bound with the DNA. The binding free energy can reflect the degree of binding between a ligand and a receptor to some extent, that is, the smaller the binding free energy, the closer the binding. The energies obtained from the molecular docking studies were consistent with our thermodynamic study. In the binding conformation, AFG1’s molecular structure attained a certain degree of translation and reversion compared with AFB1. By comparing the folding of the binding conformation of AFB1/AFG1 with the DNA (Figure 13), the two carbonyl O atoms in the AFG1 structure all approached the interior of the DNA groove during binding, whereas that of the AFB1 approached the exterior of the DNA groove. This difference can be attributed to the larger contact area between AFG1 and the DNA than between AFB1 and the DNA. The carbonyl O also belongs to the hydrogen bond acceptor, and moves toward the inside of the DNA groove. This behavior is beneficial to the formation of hydrogen bonds. All of these differences explain the variations in the above-mentioned energy analysis. Hydrogen bonding plays a significant role in DNA structure formation and the total stability of the system, and therefore the formation of hydrogen bonds has been explored [56]. Three hydrogen bonds formed between AFB1 and two DNA bases (DA-17 and DG-16) (Figure 14a). One of the hydrogen bonds was formed between the O3 atom of AFB1 and DG-16, and the others were formed between the O6 atom of AFB1 and DA-17 associated with DG-16 on the B-chain. All of the hydrogen bonds were strong between AFB1 and the DNA (distance from 2.5 Å to 3.0 Å) (Table 4). Meanwhile, AFG1 formed six hydrogen bonds with different DNA bases on the A- or B-chain (Figure 14b). These bonds included two strong hydrogen bonds, three moderate hydrogen bonds, and one weak hydrogen bond (Table 4). Among the six hydrogen bonds, three were located in the carbonyl O atom of C-20, two were formed in the carbonyl O atom of C-19, and the other was built in the O18 atom of AFB1. When hydrogen bonds were formed between AFB1/AFG1 and the DNA, the positions of AFB1 and AFG1 differed because of their molecular structures. We inferred that these positions were related to the different toxic effects of AFB1 and AFG1 in vivo. Thus, we concluded that the molecular docking results further verified that AFB1/AFG1 interacts with DNA through groove binding, which was consistent with the spectroscopic studies.

## 3. Discussion

In this work, the characteristics of the interaction between AFB1/AFG1 and ctDNA in a pH 7.4 Tris-HCl buffer were determined through different spectroscopic methods coupled with viscosity measurements, DNA melting assay, and molecular docking techniques. The fluorescence results demonstrated that ctDNA quenched the intrinsic fluorescence of AFB1/AFG1 through different quenching mechanisms. The AFG1–DNA complexation was driven mainly by hydrogen bonding and van der Waals forces. Unlike AFG1, hydrogen bonding with hydrophobic interactions played major roles in the binding of AFB1 with the DNA. The binding constant values of the AFG1–DNA complex were always larger than those of the AFB1–DNA complex even in different temperatures. Based on the denatured DNA and viscosity measurements, as well as the UV absorption spectroscopy and competitive binding study, we concluded that both AFB1 and AFG1 interacted with ctDNA via the groove binding mode. CD analysis suggested partial conformation changes on ctDNA upon AFB1’s addition and AFG1’s addition. Moreover, the results of molecular docking showed that the specific binding site mainly existed between AFB1/AFG1 and the A–T base pairs of ctDNA via the formation of hydrogen bonds.

The experimental results indicated that AFB1/AFG1 could directly bind with DNA through noncovalent interactions after AFB1/AFG1 enters into the body unmetabolized. Moreover, owing to such binding, aflatoxins influenced the DNA’s structure and even damaged the DNA. We believe that the data gathered in this study will help in understanding the mechanism of DNA damage and the toxicological effects of aflatoxins. Additionally, the results of this study also provide guidance for the construction of an in vitro digestion model. This research can serve as a basis for the revision of food safety standards, and provide a comprehensive reference on risk assessment for mycotoxins.

## 4. Materials and Methods

### 4.1. Chemicals and Reagents

The ctDNA (D1501-100 mg) was purchased from Sigma Corporation (St. Louis, MO, USA) and dissolved in phosphate-buffered saline (PBS) (pH 7.4) at 4 °C with occasional stirring to ensure the formation of a homogeneous solution. The absorbance ratio at 260/280 nm was recorded to check the purity of the DNA solution. The ctDNA’s concentration was determined by using the absorption at 260 nm and the molar absorption coefficient ε_260_ = 6600 L·mol^−1^·cm^−1^. The stock solution (4 × 10^−3^ mol·L^−1^) of AFB1/AFG1 (Sigma) was prepared by dissolving it in 95% (*v*/*v*) methanol. The ethidium bromide (EB) and Hoechst 33258 were purchased from Sigma Corporation. A total of 0.05 mol·L^−1^ PBS (containing NaH_2_PO_4_·2H_2_O and Na_2_HPO_4_·12H_2_O at pH 7.40) was used to simulate physiological conditions. The NaH_2_PO_4_·2H_2_O, Na_2_HPO_4_·12H_2_O, NaCl, and methanol were purchased from Kelong Chemical Reagent Factory (Chengdu, China). All of the other reagents were of analytical grade, and double-distilled water was used in all of the experiments.

### 4.2. Instrumentation

The UV–vis absorption spectra were recorded on a Shimadzu UV-2450 spectrophotometer (Shimadzu, Tokyo, Japan) using a 1.0× 1.0 cm quartz cell. The fluorescence and resonance light scattering (RLS) measurements were performed using a F-4500 spectrofluorometer (Hitachi, Tokyo, Japan) using a 1.0 cm quartz cell. The viscosity measurements were performed on an Ubbelohde viscometer (Φ 0.7–0.8 mm, Shanghai Qianfeng Rubber and Glass Co., Shanghai, China). The CD spectra were obtained on a MOS 450 CD spectrometer (Bio-Logic, Paris, France) using a 1.0 mm path quartz cuvette. The pH was measured using a pH S-25 digital pH meter (Leici, Shanghai, China).

### 4.3. Dialysis Assays

Dialysis bags (MWCO 1 kDa)(Sigma-Aldrich, St. Louis, MO, USA) were used for the solutions of AFB1–DNA and AFG1–DNA. The solutions, including 1.51 × 10^−4^ mol·L^−1^ DNA and 4 × 10^−5^ mol·L^−1^ AFB1/AFG1, were dialyzed for 24 h at room temperature. The UV absorbance and fluorescence emission values were recorded for the solutions in the dialysis bags.

### 4.4. Fluorescence and Resonance Light Scattering (RLS) Spectra

The fluorescence quenching spectra were obtained in the range of 300–700 nm at an excitation wavelength of 365 nm. The fluorescence spectra were obtained at three temperatures (298, 303, and 308 K). The excitation and emission slits were set at 5 nm.

A 1.0 mL solution containing 8 × 10^−6^ mol·L^−1^ AFB1/AFG1 was added to the quartz cuvette and then titrated through successive additions of ctDNA (concentration range from 1.6 × 10^−6^ mol·L^−1^ to 16 × 10^−6^ mol·L^−1^). Then, the solutions were mixed sufficiently and allowed to stand for 10 min to achieve equilibrium. The RLS spectra were obtained through synchronous scanning (Δ*λ* = 0 nm) with a wavelength range of 200−700 nm at room temperature. Both the excitation and emission slits were set at 5 nm.

### 4.5. Competitive Displacement Assays

The displacement assays of Ethidium bromide (EB) and Hoechst 33258 were performed with a solution containing a fixed concentration of EB/Hoechst 33258 and ctDNA (2 × 10^−6^ mol·L^−1^ and 4 × 10^−5^ mol·L^−1^). Then, the solution was titrated by increasing the AFB1/AFG1 concentrations (from 0 to 8 × 10^−5^ mol·L^−1^). The EB–DNA mixture was excited at 515 nm, and the emission spectra ranged from 400 nm to 700 nm. Meanwhile, the DNA–Hoechst 33258 complex was excited at 340 nm, and the emission spectra ranged from 300 nm to 700 nm.

### 4.6. UV–Visible Spectroscopy

The absorption spectra of AFB1/AFG1 and the AFB1/AFG1–DNA complexes were obtained in the wavelength range of 190–500 nm. The experiments were performed by using a fixed small-molecule concentration (3.5 × 10^−5^ mol·L^−1^) while varying the ctDNA concentration from 0 to 25.6 × 10^−6^ mol·L^−1^. Another experiment involved a fixed DNA concentration (2 × 10^−5^ mol·L^−1^) while varying the ctDNA concentration from 0 to 32 × 10^−6^ mol·L^−1^.

### 4.7. Viscosity Measurements

The viscosity assay of the fixed ctDNA solution (2 × 10^−5^ mol·L^−1^) was performed in the absence and presence of AFB1/AFG1 at various concentrations (from 0 to 2.8 × 10^−5^ mol·L^−1^). All of the solutions were thermostated at 25 ± 0.1 °C through a thermostatic bath. The flow times of the different solutions through the capillary were measured in three replicates using a digital stopwatch. The data were presented as (η/η_0_^)1/3^ versus the ratios of the concentration of ([AFB1/AFG1]/[ctDNA]), where η and η_0_ represent the viscosity of ctDNA in the presence and absence of AFB1/AFG1 [16]. The viscosity values were determined from the formula η = (*t*−*t*_0_)/*t*_0_, where t and t_0_ are the observed flow time of ctDNA-containing solutions and buffer solutions [16].

### 4.8. DNA Melting Assay

The melting-temperature (*T*_m_) experiment was recorded in a thermostat water bath from 20 °C to 100 °C, with 5 °C increments. This part of the experiment was studied in the absence and presence of AFB1/AFG1 by measuring the absorbance intensity of ctDNA solutions at 260 nm. *T*_m_ was obtained from the transition midpoint of the thermal denaturation curves based on fss versus temperature (*T*), that is, *f*_ss_ = (*A* − *A*_0_)/(*A*_f_ − *A*_0_), where *A*_0_ is the initial absorbance value, *A* is the absorbance intensity corresponding to the temperature, and *A*_f_ is the final absorbance value [57].

### 4.9. Effect of Ionic Intensity

The effect of ionic intensity was determined by monitoring the change of fluorescence intensity of the systems. To prepare the systems, different NaCl concentrations ranging from 5 mol·L^−1^ to 45 × 10^−3^ mol·L^−1^ were added to the solution containing 2 × 10^−5^ mol·L^−1^ AFB1/AFG1 and 2 × 10^−5^ mol·L^−1^ ctDNA. The excitation and emission wavelengths were set to 365 nm and 440 nm.

### 4.10. Interaction with Single-Stranded ctDNA (ss-ctDNA) and Double-Stranded ctDNA (ds-ctDNA)

The single-stranded ctDNA (ss-ctDNA) was obtained by heating double-stranded ctDNA (ds-ctDNA) in a boiling water bath for 30 min and then rapidly cooling it in an ice water bath for 8 min [58]. To compare the fluorescence quenching effect of ssDNA and dsDNA on the AFB1/AFG1 intensity, ssDNA or dsDNA solution was successively added to the AFB1/AFG1 solution. Then, the corresponding quenching constants were calculated and compared.

### 4.11. Circular Dichroism (CD) Spectroscopy

The CD spectra of the solutions containing fixed ctDNA concentrations (1 × 10^−5^ mol·L^−1^) with varying AFB1/AFG1 concentrations (0, 4 × 10^−6^, and 20 × 10^−6^ mol·L^−1^) were obtained at wavelengths between 200 nm and 500 nm. All of the CD spectra were scanned every 1 nm for four times with a speed of 100 nm·min^−1^. The baseline of the buffer solution (0.05 mol·L^−1^ PBS, pH 7.4) of the CD signals was deducted from each spectrum. 

### 4.12. Molecular Docking

The molecular docking experiments were performed with Auto Dock 4.2.6 software. The crystal structures of AFB1 and AFG1 were obtained from the Pubchem database. . With the aid of Auto Dock tools, polar hydrogen atoms and Gasteiger charges were added. The ligand roots of AFB1 and AFG1 were also detected, and the rotatable bonds were defined. The structure of B-DNA dodecamer 5′–d(CGCGAATTCGCG)2–3′ (PDB ID:1BNA) was available in the Protein Data Bank (PDB) [59]. This structure was further modified for docking calculations through Auto Dock tools. All of the water molecules were removed, whereas the Gasteiger charges and essential hydrogen atoms were added. The docking programs were run using Lamarckian Genetic Algorithms to create an interaction between AFB1/AFG1 and DNA. For docking calculations, to cover the entire binding site of DNA and provide space for the ligands to move freely, a grid box of 95 × 102 × 139 points with a grid spacing of 0.375 Å was prepared. All of the other parameters were defined, and the default values were given by Auto Dock. The grid energy of the docking pocket area was calculated by an AutoGrid program. A total of 100 GA runs were accomplished, and the best structure with the lowest binding free energy was used.

### 4.13. Statistical Analysis

The results were expressed as mean ± SD of at least three independent observations. Origin 9.0 software (OriginLab Corp., Northampton, MA, USA) was used for the background correction of the fluorescence emission spectra and the evaluation of the fluorescence intensities.

In this paper, all of the fluorescence data has been subject to inner filter correction in order to eliminate any inner filter effects.

## Figures and Tables

**Table 1:** The Stern-Volmer quenching constants and association constants of AFB1/AFG1 quenched by ctDNA at different temperatures.

Aflatoxins	*T* (K)	*K_sv_* ^a^ (L·mol^−1^)	*R* ^b^	*K_a_* ^c^ (L·mol^−1^)	*R* ^d^
AFB1	298	1.24 × 10^4^	0.9938	4.12 × 10^3^	0.9921
	303	2.31 × 10^4^	0.9959	4.97 × 10^3^	0.9901
	308	3.22 × 10^4^	0.9902	5.48 × 10^3^	0.9881
AFG1	298	8.31 × 10^4^	0.9912	1.89 × 10^4^	0.9865
	303	7.32 × 10^4^	0.9887	1.27 × 10^4^	0.9908
	308	0.55 × 10^4^	0.9908	9.82 × 10^3^	0.9918

^a^ The Stern–Volmer constant; ^b^ The correlation coefficient of the *K_sv_* values; ^c^ The binding constant; ^d^ The correlation coefficient of the *K_a_* values.

**Table 2:** Relative thermodynamic variables for the interaction of AFB1/AFG1 with ctDNA at different temperatures.

Systems	Δ*H* ^a^ (kJ·mol^−1^)	Δ*S* ^b^ (J·mol^−1^·K^−1^)	Δ*G* ^c^ (kJ·mol^−1^)
AFB1-ctDNA	−24.45	17.88	−31.47
			−31.98
			−32.56
AFG1-ctDNA	−27.54	−19.34	−36.32
			−35.97
			−35.65

^a^ Enthalpy changes; ^b^ Entropy change; ^c^ Gibbs free energy.

**Table 3:** Binding energies of aflatoxins and DNA according to molecule docking result.

Complex	Intermolecular Energy (kcal·mol^−1^)	Internal Energy (kcal·mol^−1^)	Torsional Energy (kcal·mol^−1^)	Unbound Extended Energy (kcal·mol^−1^)	Lowest Binding Energy (kcal·mol^−1^)	Electrostatic Energy (kcal·mol^−1^)
AFB1-DNA	−8.08	−0.12	0.30	−0.12	−7.78	−0.46
AFG1-DNA	−8.59	−0.09	0.30	−0.09	−8.29	0.04

**Table 4:** The strength of hydrogen bonds in the interaction of AFB1/AFG1 with DNA by molecule docking.

Small Molecular	Strong Hydrogen Bonds	Moderate Hydrogen Bonds	Weak Hydrogen Bonds
AFB1	2.6 Å (DG-16) 2.8 Å (DA-17) 2.9 Å (DA-17)		
AFG1	2.9 Å (DT-7) 2.9 Å (DT-8)	3.1 Å (DT-19) 3.2 Å (DA-18) 3.4 Å (DT-19)	3.6 Å (DT-20)
